# Water Supply and Health

**DOI:** 10.1371/journal.pmed.1000361

**Published:** 2010-11-09

**Authors:** Paul R. Hunter, Alan M. MacDonald, Richard C. Carter

**Affiliations:** 1 School of Medicine, Health Policy and Practice, University of East Anglia, Norwich, United Kingdom; 2 British Geological Survey, Edinburgh, United Kingdom; 3 WaterAid, London, United Kingdom

## Abstract

As one article in a four-part *PLoS Medicine* series on water and sanitation, Paul Hunter and colleagues argue that much more effort is needed to improve access to safe and sustainable water supplies.

**Summary Points**

- A safe, reliable, affordable, and easily accessible water supply is essential for good health, but for several decades almost 1 billion people in developing countries have lacked access to such a supply.
- A poor water supply impacts health by causing acute infectious diarrhoea, repeat or chronic diarrhoea episodes, and nondiarrhoeal disease, which can arise from chemical species such as arsenic and fluoride. It can also affect health by limiting productivity and the maintenance of personal hygiene.
- Reasons for the limited progress towards universal access to an adequate water supply include high population growth rates in developing countries, insufficient rates of capital investment, difficulties in appropriately developing local water resources, and the ineffectiveness of institutions mandated to manage water supplies (in urban areas) or to support community management (in rural areas).
- Strenuous efforts must be made to improve access to safe and sustainable water supplies in developing countries, and, given the health burden on the public and the costs to the health system, health professionals should join with others in demanding accelerated progress towards global access to safe water.

 **This is one article in a four-part** ***PLoS Medicine*** **series on water and sanitation.**

## Introduction

A safe, reliable, affordable, and easily accessible water supply is essential for good health. Yet, for several decades, about a billion people in developing countries have not had a safe and sustainable water supply. It has been estimated that a minimum of 7.5 litres of water per person per day is required in the home for drinking, preparing food, and personal hygiene, the most basic requirements for water; at least 50 litres per person per day is needed to ensure all personal hygiene, food hygiene, domestic cleaning, and laundry needs [1]. This domestic water consumption is dwarfed by the demands of agriculture and ecosystems, even in wealthy countries where per capita domestic water consumption greatly exceeds these figures [2]. To cover all these requirements and to avoid water stress, experts generally agree that about 1,000 cubic metres of freshwater per capita per year is needed [3].

A key target of Millennium Development Goal (MDG) 7, which aims to ensure environmental sustainability, is “to reduce by half the proportion of people without sustainable access to safe drinking water and basic sanitation by 2015” [4]. This water supply target underpins several other MDGs, including those relating to poverty (MDG1), education (MDG2), and gender equality (MDG3). In particular, it underpins MDG4, the reduction of child mortality, because many deaths in young children in developing countries are due to diarrhoeal disease, and unsafe water is a key risk factor for diarrhoeal disease in this age group [5].

The WHO/UNICEF Joint Monitoring Programme for Water-supply and Sanitation (JMP), which monitors progress on the MDG water supply target, identifies three categories of drinking water supply: (a) water piped into the dwelling, plot, or yard; (b) other improved sources (including public taps, protected springs, hand pumps, and rainwater harvesting); and (c) unimproved sources (open water, unprotected from contamination) [6]. JMP assumes that “improved” water should be available not only for drinking but also for food preparation and personal and home hygiene, but it provides no official definition of how near a water source should be to a dwelling to be called improved. However, a distance of <1,000 m has been suggested as an appropriate distance for meeting the MDG targets [7].

In poorly served countries, achieving the MDG water supply target will involve increasing water availability for domestic uses, improving water quality, and bringing about changed water-use and water-management habits. In the wealthy countries where adequate quantities of domestic water are already available on demand, the main task over the next few years will be to sustain water quality given the increasing pressures of pollution. However, global water supply targets need to be tempered by a recognition of the real demand (as expressed in user willingness and ability to pay), which may be less ambitious than the internationally agreed target. Furthermore, account needs to be taken of the realities of frequently poor levels of functionality. It is relatively easy to increase coverage through construction of water supply systems, but it is much more difficult to ensure that such systems continue to provide service over the long term.

We therefore argue in this paper for a serious commitment by national governments and their partners to ensure adequate water supply services for all (the MDG target, if met, would still leave 672 million people with an unimproved supply [6],[8]). In addition, we call for increased attention to be paid to ensuring continuing service provision. This will mean finding new ways to enhance public demand for improved services (that might translate into a willingness to pay), and a public and private sector ethos that puts high value on the quality of construction and ongoing service delivery.

## Water Supply and Health

Inadequacies in water supply affect health adversely both directly and indirectly (Box 1 and below). An inadequate water supply also prevents good sanitation and hygiene. Consequently, improvements in various aspects of water supply represent important opportunities to enhance public health. Box 2 lists six attributes of domestic water supply that determine whether it is effective in the preservation of good health [14].

**Box 1. The Classification of Water-Related Disease**

The standard classification of water-related disease was first proposed by David Bradley [9] (Table 2). Although there have been suggested improvements since [10], none have gained as much recognition as the original system, probably because they are less focused on disease transmission mechanisms.

Although this four-part classification has served a useful role in highlighting some of the public health impacts of inadequate water systems, it has also directed attention away from some other important health issues, namely:

- There is no room in the classification for chemical-mediated diseases such as arsenic and fluoride poisoning, which have major impacts in certain localities [11],[12].
- The classification takes no account of the impact on health of the need to collect and transport water for many of the world's population. For example, many children and women in developing countries have to carry heavy containers of water long distances each day, and there have been no systematic studies about the impact this has on musculoskeletal health.
- The long walks needed to collect water may also increase the spread of certain infectious diseases though a community. For example, an epidemic of meningococcal disease in a Sudanese refugee camp seemed to spread along the routes that people take to collect their water [13].
- Unpleasant tastes or odours (for example, arising from iron content of groundwater, or associated with chlorination) in water supplies that are microbiologically safe may act as a deterrent to use of safe sources, so exposing users to health risks associated with unprotected water sources.

**Box 2. Six Factors That Determine Whether a Water Supply Can Maintain Good Health Effectively**

*The quality* of the water relates to pathogens and chemical constituents in water that can give rise to both diarrhoeal and nondiarrhoeal disease.

The *quantity* of water available and used. This is largely determined by (a) the distance of carry involved, where water has to be transported (often on the heads or backs of children and women), and (b) the wealth of the user.

*Access* to water may be primarily a matter of physical distance or climb, but it may have socioeconomic and/or cultural dimensions if certain social groups are denied access to particular water sources through cost or culture (see Figure 4).

The *reliability* of both unimproved and improved water supplies. Many cities in Asia, for example, supply piped water for only a few hours per day, or for a few days in every week and many unimproved rural water supplies dry up regularly.

The *cost* of water to the user. This is represented by the cash tariff that is paid to a utility or provider or, in the case of unimproved water supplies, by the time and health penalty paid by the user.

The *ease of management* for the end user. In urban utility-managed supplies the user merely pays a tariff; in rural settings in developing countries, users are expected to play a major part in operation, maintenance, and management.

### Water, Diarrhoea, and Infant Mortality

**Figure 1:**
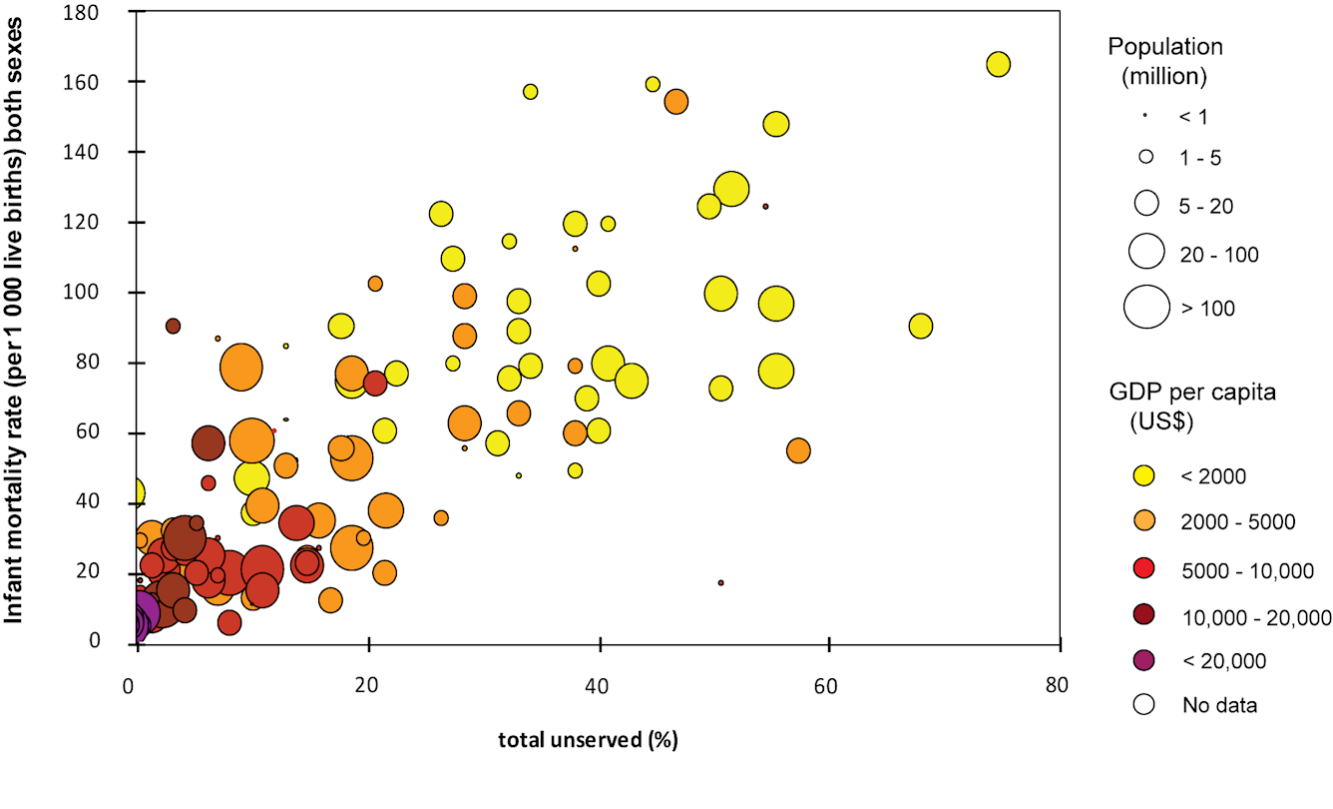
Global association between national access to improved water source, GDP and infant mortality. Data sources [6],[20],[66].

**Figure 2:**
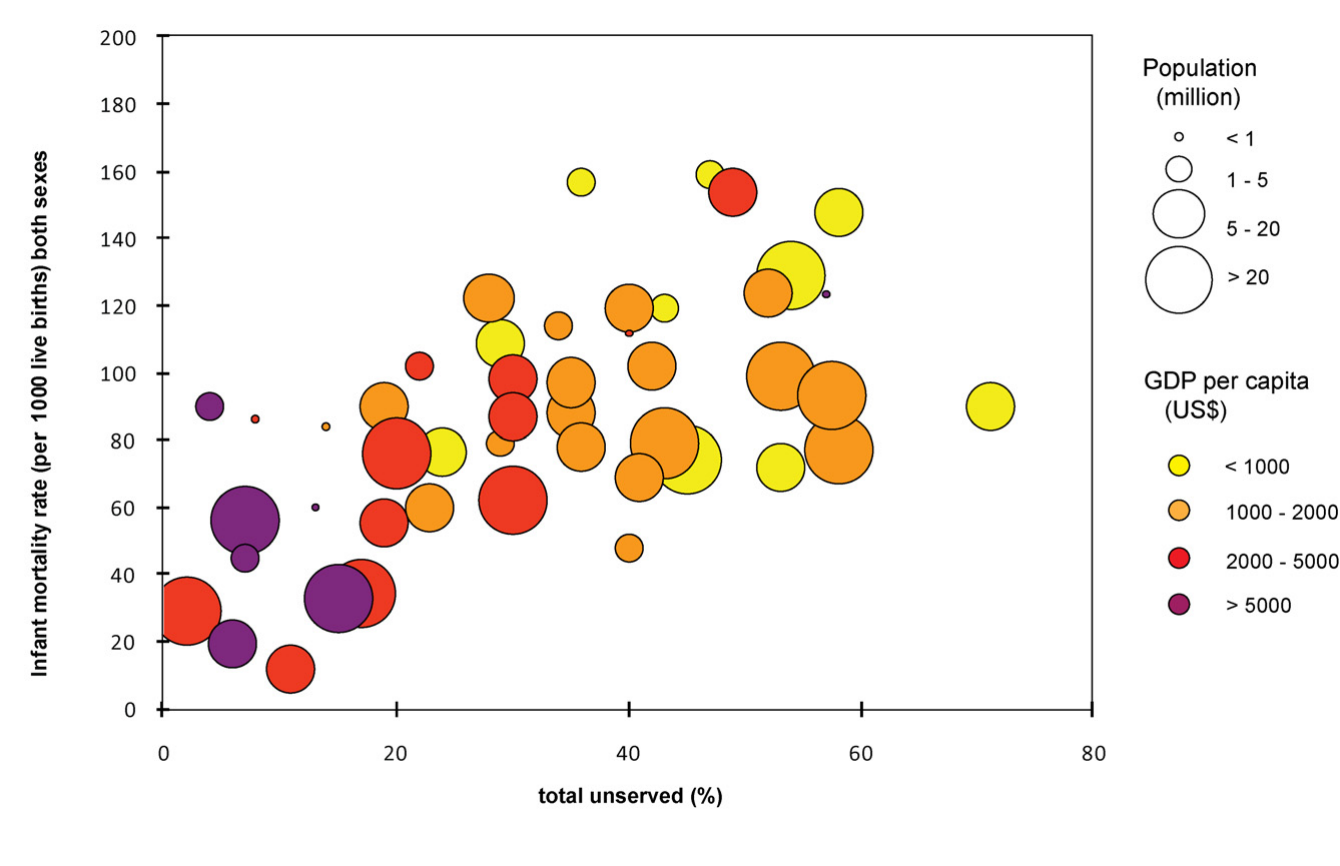
Association between national access to improved water source, GDP and infant mortality for Africa. Data sources [6],[20],[66].

Investigations of the costs and health benefits associated with improvements to drinking water supply in low-income countries have concentrated almost exclusively on how these improvements affect the incidence of acute infectious diarrhoea [15]–[17]. This focus is not surprising given that diarrhoeal disease is the second most common contributor to the disease burden in developing countries (as measured by disability-adjusted life years [DALYs]), and poor-quality drinking water is an important risk factor for diarrhoea [18],[19]. Most of the excess disease burden in developing countries falls on young children—17% of all deaths in children under 5 years are attributed to diarrhoea [15]. Figures 1 and 2 illustrates how an inadequate water supply is a contributor to deaths in children under 5 years [18],[19]. It shows that both the gross domestic product per capita (GDP) [20] and the proportion of the population without access to improved water are highly correlated with infant mortality (*p*<0.001 for both). Both measures remain independent risk factors for infant mortality in a multiple predictor variable regression. While this analysis does not prove a direct causal relationship, since access to improved water services is likely to be accompanied by improvements in other services (such as sanitation), it is clear that a broad statistical relationship exists between improved water services and lower infant mortality for countries of similar GDP.

The focus on acute diarrhoea, however, almost certainly underestimates the disease burden caused by inadequate water and sanitation. There is a strong link between repeat or chronic diarrhoeal disease, malnutrition, and the poor educational and physical growth that can seriously affect the ability of children to reach their full potential [21]. It has been suggested that if the impacts of these chronic effects are taken into consideration, the real global disease burden due to diarrhoea (and, consequently, the health benefits of water and sanitation interventions) would be about twice the current estimates, which are based only on acute illness and mortality [21].

The evidence that improving access to safe drinking water reduces the risk of diarrhoeal disease in children is strong. However, since the early 1980s and especially since Esrey's work in the late 1980s [22], there has been a heated debate over the relative importance of water quantity and water quality in reducing the incidence of diarrhoeal disease. The rather differing analyses of Esrey and subsequent workers have led to different emphases in water supply interventions—especially in regard to the role of point-of-use household water treatment technologies (see Text S1).

Importantly, however, whatever intervention is introduced, recent evidence suggests that even occasional short-term failures in water supply or water treatment can seriously undermine many of the public health benefits associated with an improved water supply [23]. This evidence is not an argument against attempting to improve water quality, whether through community or household water treatment technologies, but it draws attention to the vital importance of developing systems that will continue to deliver safe water in the long term.

### Drinking Water and Nondiarrhoeal Disease

Inadequate access to safe drinking water is also associated with several nondiarrhoeal diseases [24]. Chronic or acute exposure to many organic and inorganic chemical agents has been implicated in adverse health effects that range from acute nausea and vomiting or skin rashes, to cancer and foetal abnormalities [24]. Inorganic pollutants in drinking water that have been linked with disease include arsenic, copper, fluoride, lead, and nitrate. Organic compounds that have caused concern include pesticides, chlordane, phenol, and trihalomethanes [24]. More recently, endocrine-disrupting compounds and pharmaceuticals in drinking water have been causing concern [25].

In the developing world, one of the most dramatic demonstrations of the link between drinking water and nondiarrhoeal disease is the arsenic crisis in Bangladesh [26]. Arsenic in drinking water can have substantial adverse effects on health, including skin cancer and gangrene [27]. The Bangladesh crisis occurred because boreholes constructed to provide people with clean drinking water often provided water with naturally high arsenic concentrations. Fluoride in drinking water is also causing increasing concern in the developing world. About 200 million people are at risk of exposure to elevated concentrations of fluoride in drinking water, which can lead to dental and sometimes skeletal fluorosis [12],[28]. Although the global disease burden estimates for nondiarrhoeal diseases associated with water supply problems such as these fall far below similar estimates for diarrhoeal disease [29],[30], the communities actually affected by these diseases can suffer severely.

### Indirect Links between Water and Health

In addition to the direct health benefits of improved safe water supplies, there are many indirect benefits. For example, the strong relationship between water and livelihoods in all regions and economies of the world affects health indirectly. In developing countries, deficiencies in water supply, whether for productive or domestic uses, have direct negative impacts on livelihoods; in wealthier countries, past investment in water infrastructure and the ability to invest more in the present increase water security and, arguably, prosperity [31].

Lack of water can also lead indirectly to disease via malnutrition. Several authors argue strongly for investments in low-cost water harvesting techniques, irrigation, and clean water provision as a means of increasing food production and reducing infectious disease burden [32],[33]. Numerous examples exist across sub-Saharan Africa and south Asia in which access to a small amount of irrigated land has transformed food security for highly vulnerable households [34]. A study of child nutrition in otherwise comparable communities with and without access to irrigation in central Kenya found clear evidence that irrigation contributed to higher energy intakes and reduced chronic malnutrition in children [35]. However, mixed conclusions were found in a study comparing households close to and distant from two dams in Burkina Faso [36].

Finally, improvements in water supply are essential prerequisites for improved personal and home hygiene and to enable sanitation facilities to be kept clean. Consequently, the direct health effect of improved water supply is likely to be extended by its indirect effects on sanitation and hygiene.

## Economic Returns

A recent study of the economic returns on investments in water supply and sanitation indicated that every US$1 spent on water supply and sanitation services could lead to an economic return of between $5 and $46, with the highest returns in the least-developed areas [16]. Much of this additional income was from the time saved by having reliable water close to the household. Other studies also suggest that investments in water alleviate poverty [37],[38]. The balance of evidence favours the likelihood that water and sanitation interventions have economic benefits beyond those that simply relate to reduced health care costs. Indeed, it has been argued that adequate water and sanitation is an essential prerequisite to economic development. Thus, poor countries with access to improved water experienced average annual growth of 3.7% whereas countries with the same per capita income but without such access have an annual growth of only 0.1% [39].

### Status and Trends

Nowadays, many more people have an improved water supply, as defined by JMP [6], than in the late 1970s. However, this increased coverage has only just matched global population growth. The absolute number of people lacking access to an improved water supply has hovered around 1 billion since the late 1970s [6],[40]. Unfortunately, it is probable that the populations remaining to be adequately served (for example, in remote rural areas of low-income countries and in the periurban slums of the world's towns and cities) represent the most intractable problems.

The 1990 (base year for the MDGs) and 2008 (most recent) statistics on urban water supply show coverage rising from 95% to 96%, while the total urban population has grown over this period from 2.3 billion to 3.4 billion. In rural areas, the coverage estimates are 64% and 78% for 1990 and 2008, respectively, while the total rural population has grown from 3.0 billion to 3.4 billion (Table 1). Overall, 84% of people still not enjoying an improved water supply live in rural areas, but it is the urban areas that are struggling most to keep ahead of population growth rates, which are commonly double the national averages.

**Table 1:** Use of urban, rural and total improved water, 1990, 2000, and 2008, globally and regionally.

MDG Region	Year	Population (Thousands)	% Urban Population	Use of Improved Drinking-Water Sources (% of Population)		
				Urban	Rural	Total
Sub-Saharan Africa	1990	517,961	28	83	36	49
	2000	674,693	33	82	42	55
	2008	822,436	37	83	47	60
Northern Africa	1990	120,675	49	94	78	86
	2000	144,621	51	94	83	89
	2008	164,466	53	95	87	92
Eastern Asia	1990	1,213,509	30	97	56	69
	2000	1,345,739	38	98	70	81
	2008	1,419,532	45	98	82	89
Southern Asia	1990	1,200,043	26	91	69	75
	2000	1,462,960	29	93	76	81
	2008	1,668,746	31	95	83	87
South Eastern Asia	1990	439,591	32	92	63	72
	2000	517,193	40	92	72	80
	2008	575,626	47	92	81	86
Western Asia	1990	135,850	61	96	70	86
	2000	174,394	65	96	74	88
	2008	207,991	67	96	78	90
Oceania	1990	6,449	24	92	38	51
	2000	8,121	24	92	40	52
	2008	9,633	23	92	37	50
Latin America and the Caribbean	1990	442,310	71	95	63	85
	2000	521,228	75	96	72	90
	2008	576,102	79	97	80	93
Commonwealth of independent states	1990	280,899	65	98	82	92
	2000	280,998	64	98	84	93
	2008	276,820	64	98	87	94
Developed regions	1990	933,073	71	100	98	99
	2000	985,273	74	100	98	100
	2008	1,028,520	75	100	98	100
Developing regions	1990	4,076,387	35	93	60	71
	2000	4,848,948	40	94	69	79
	2008	5,444,533	44	94	76	84
World	1990	5,290,359	43	95	64	77
	2000	6,115,219	47	96	71	83
	2008	6,749,872	50	96	78	87

Source: [2].

**Table 2:** Water-related disease.

Category	Description	Example Diseases
Waterborne disease	Enteric infections spread through faecal contamination of drinking water	Typhoid, *Campylobacter*, giardiasis, *Cryptosporidium*, cholera, enterohemorrhagic and enterotoxigenic *E. coli*, norovirus, etc.
Water-washed diseases	Infections that spread in communities that have insufficient water for personal hygiene	Trachoma, scabies, *Shigella*
Water-based diseases	Diseases where the causative organism requires part of its lifecycle to be spent in water	Schistosomiasis, dracunculiasis
Water-related diseases	Vector-borne diseases where the insect vector requires access to water	Malaria, onchocerciasis, trypanosomiasis

Source: [9].

With 2015 (the MDG target year) fast approaching, there is a heavy emphasis internationally on accelerating progress towards the coverage target. Nevertheless, this push needs to be tempered with realism and an emphasis on maintaining existing water supplies in a functional state. No-one wishes to see developing countries littered with defunct water supply systems as a legacy of the MDGs.

### Delivering a Better Water Supply

In wealthy nations, high-quality water is universally available with large amounts of money being spent to assure reliable household supplies. In poorer countries, improved access to water is generally delivered through communally managed public water points in rural areas and unreliable distribution systems in towns and cities. Unfortunately, many water supply interventions in developing countries do not last [41]. In a recent study of 15 villages in South Africa with supposedly improved water supplies, three villages had insufficient water because their wells had dried up or were incapable of meeting the demand [42]. Five more villages had no water on the day of inspection—two because their water pump had broken, two because there was no money to buy diesel for the pump, and one because the pump operator was ill. This example illustrates some of the challenges associated with keeping water supply systems working over the long term. Similarly, a study of water supplies and arsenic mitigation technologies in Bangladesh found that only about 64% of the interventions were operational [43]; other studies suggest that across sub-Saharan Africa about one third of hand pumps are nonfunctioning [44].

Unfortunately, in low-income countries, revenues recovered from the users of improved water supplies are frequently insufficient to meet the real running costs of both rural and urban water supplies, so systems either deteriorate or need to be heavily subsidized. It is important that user tariffs are affordable, but that promising approaches for improving revenue generation include finding ways to reduce or spread the costs of establishing house connections (in the case of urban piped supplies), developing microfinance instruments for rural user fees, and encouraging self-help and small enterprise-driven approaches. Nevertheless, there needs to be recognition that the true demand for improved services (expressed as willingness to pay) may not yet match the level of service being promoted through international targets such as those included in the MDGs.

There is increasing recognition of the part that self-help (self-supply) initiatives and small enterprises can play in delivering improved and sustainable water services. A recent review of water, sanitation, and hygiene for the Bill & Melinda Gates Foundation identified three broad approaches to service provision: (a) externally driven approaches (initiated by agencies other than the water users, and usually heavily subsidized); (b) self-supply initiatives (driven by user demand); and (c) enterprise-driven approaches, in which local private entities supply goods and services to governments, nongovernmental organizations (NGOs), and water users directly [45]. The last two, which could be combined, represent very different approaches from the conventional, externally driven approach. However, a heavy dependence on “private” (mostly shallow groundwater) sources, which may be poorly constructed and vulnerable to contamination or failure during dry periods, has important health implications [46].

Over the last decade, debates about private sector participation and public–private partnerships for the improvement of water supply services have generated more heat than light. There is little doubt now that the private sector is unlikely to invest significant sums to modernize or extend water supply systems. However, this sector has always had an important role in the supply of goods and services, and in consultancy, supervision, and capacity-building. These roles are unlikely to disappear and we therefore take a pragmatic attitude to the involvement of the private sector: local context determines what arrangements work best [47].

In recent years, WHO has promoted the idea of Water Safety Plans (WSPs) [48]. A WSP is a risk-based approach to public health achieved through water quality and catchment management strategies under the slogan “managing drinking water quality from catchment to consumer.” Although the WSP approach is widely utilised in urban piped supply systems, there have been few attempts to implement the approach in rural settings, where distant water sources are the norm [49],[50].

## Constraints and Challenges

### Why the Slow Progress?

Slow progress toward full water supply coverage at a national level [6] may be related to national GDP [20], government effectiveness [51], or shortages of water [52]. We have explored the relationship between these three variables and coverage by statistically analysing the most recent available global datasets [6],[20],[51],[52]. Unsurprisingly, given the small amounts of water needed for domestic use, the national availability of water resources was unimportant for water supply coverage. Its significance more locally is considered below. However, the proportion of people with access to safe water was correlated with GDP (p<0.001) and government effectiveness (p<0.001). In a multivariate model, GDP remained the only significant independent covariate. Clearly, therefore, a low GDP is a major challenge facing efforts to improve water supplies. Below we discuss some of the other reasons for slow progress.

### Government Effectiveness

Government effectiveness in low-income countries is often poor, and governments often lack capacity or show institutional weaknesses [53]. Such weaknesses range from lack of individual professional skills, understaffing, poor motivation, inadequate resources, and poor organisational management, through to inappropriate policies handed down to local government from central authorities. In addition, corruption has been highlighted as a major threat to service delivery [39].

Limited effectiveness of the Ministries and local government authorities responsible for water supply can be exacerbated by insufficient political commitment at the highest governmental levels and by the weaknesses of private companies contracted to carry out construction or system management. Furthermore, the professional and technical staff of central government and local authorities often find their own high levels of commitment constrained by the systems within which they work.

### Dissociation of the Health and Water Sectors

In industrialised countries, much of the early drive to provide water and sanitation came from the medical community [54],[55]. These days, the responsibility for the management of these services usually rests with engineers and others not formally part of the public health system. This dissociation of responsibility for water services from generic public health has led to problems. For example, although benefits are usually “accrued” by the health service, the costs of water infrastructure and maintenance are borne by water utilities or boards, making expenditure decisions difficult [56]. Nevertheless, the public health community in general and the public health consultant in particular must be intimately involved in the provision of water services, playing such important roles as setting the health-based targets of WSPs [57] and designing and managing surveillance systems for waterborne disease [58].

The Walkerton tragedy—a fatal waterborne disease epidemic in Ontario, Canada that occurred in 2000—provides a good example of what can go wrong if public health oversight is completely removed from water providers [59]. One of the underlying problems in this outbreak was that the plant operators did not understand the importance and significance of water quality monitoring, so did not monitor adequately nor report problems when they occurred. These lapses led to seven deaths and an estimated 2,000+ illnesses.

### The Availability of Water Resources

**Figure 3:**
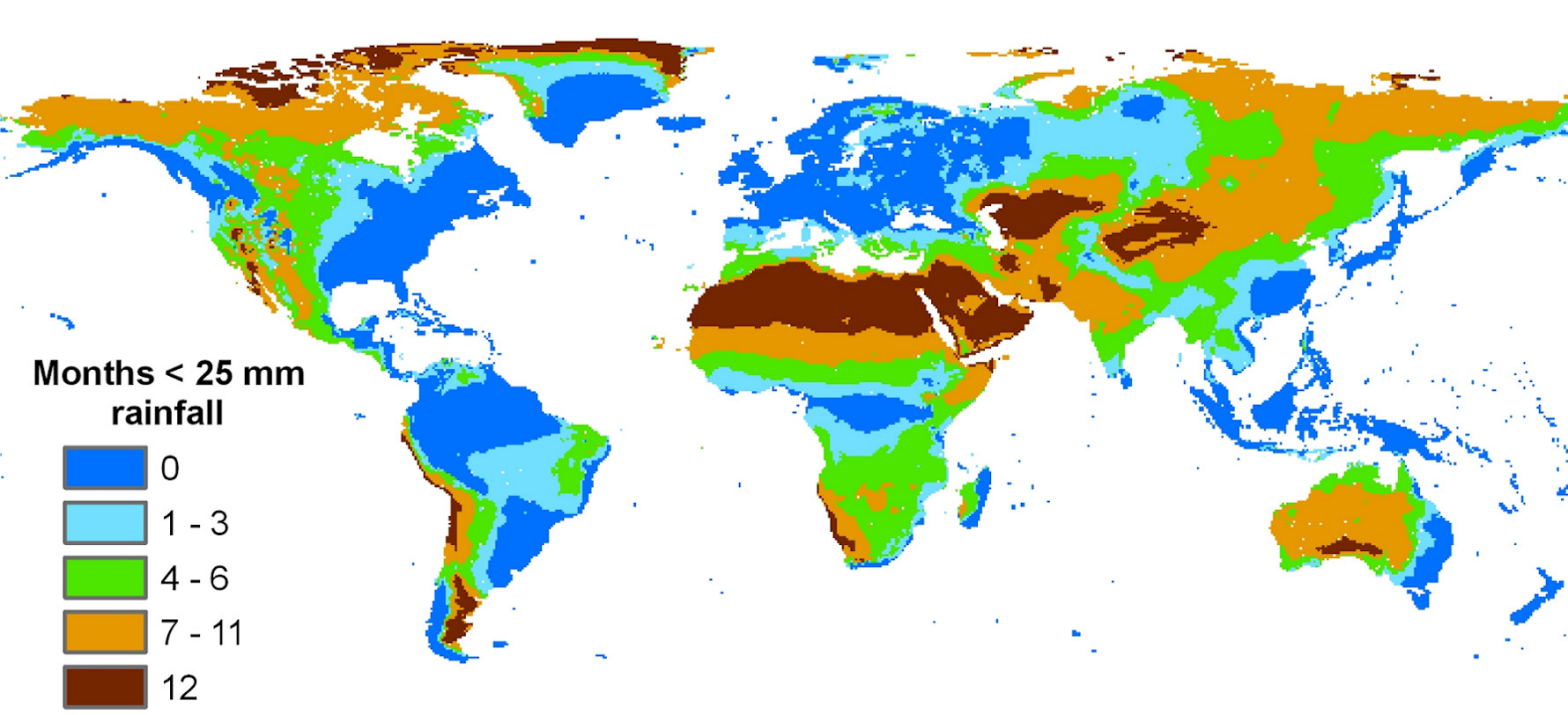
Global distribution of rainfall: The number of dry months in a year. Data source [67].

Sustainable domestic water supplies depend on the availability of reliable water resources that can be easily developed. Fresh water resources are not spread evenly across the globe (Figure 3). Most of the wealthier areas of the world experience sufficiently frequent rainfall to replenish rivers, reservoirs, and aquifers reliably, and have the capacity to store and transfer that water [31]. Nevertheless, even wealthy countries are not free from the problems of occasional droughts, as recently seen in Spain and Australia.

**Figure 4:**
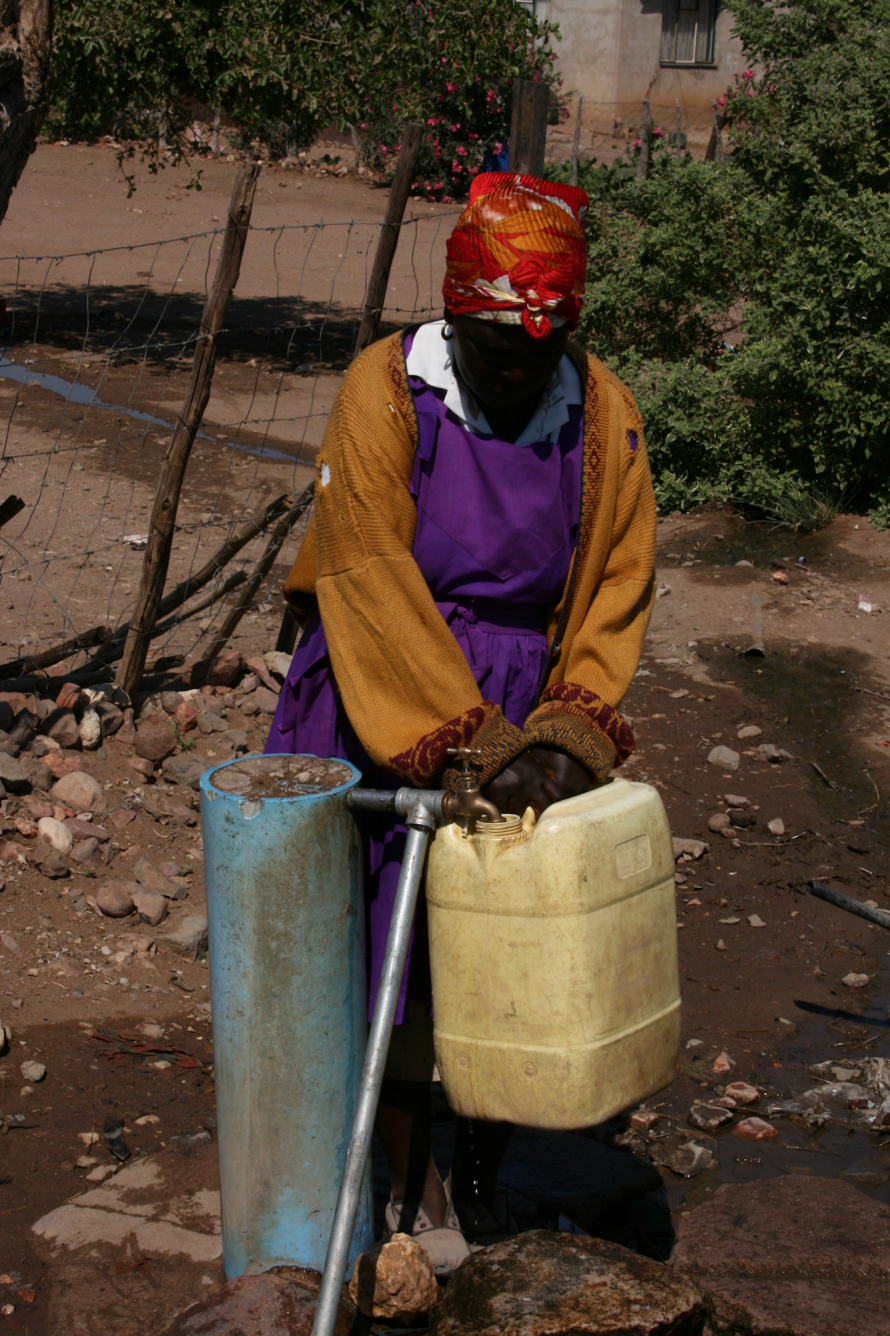
Although classified as an improved water supply, collecting water from a community tap still requires considerable time and effort. (Photograph: Paul R. Hunter).

In many parts of Africa and Asia, the long dry season and dispersed nature of many of the populations who currently have no reliable water supply mean that the development of groundwater (a natural reservoir) is the only realistic option for significantly improving drinking water coverage [60]. Consequently, statistics on *national* water resources are not a good indicator of water scarcity for much of the global population. The important factor is the availability of water resources (usually groundwater) close to the point of need. Groundwater is not a panacea, however, and its development and use need careful attention. First, in some locations even small-scale groundwater supplies can be difficult to find and develop [61]. Such locations are often a priority for water supply intervention since they are beset with diseases related to high dependence on contaminated surface water sources. A lack of appreciation of the variability in the nature and occurrence of water resources is a major reason for expensive and unreliable supplies [62].

Second, groundwater resources rely on rainfall for renewal and are strongly affected by climate variability and climate change [63]. Overabstraction of water, which can lead to falling water levels and the exhaustion of resources, is a growing global problem, exacerbated by climate change, population growth, and urbanisation (see Text S2) [64].

Finally, ground water sources across the globe are increasingly being polluted through intensive agriculture, industry, and poor sanitation [64]. For wealthy countries, this increases the costs of providing access to safe water, because more extensive water treatment is required. In poor countries, expensive water treatment is not affordable and there is little option but to drink increasingly contaminated water.

### Management of Water Supply Technology

One of the myths of community water supply in rural areas of low-income countries is that users benefiting from access to modern technology will, after a short period of training, manage the system themselves. The reality is that years of external support may be needed to build the necessary capacity [41]. Without ongoing external support (which is often absent in the context of weak local government), communities often fail to effectively manage modern technology for more than a few years.

Ever since Schumacher's seminal work promoting the idea of “intermediate technology,” individuals and organisations engaged in poverty alleviation have struggled to define what is now called “appropriate technology” [65]. The key is the match, or “fit,” between the technology, the users, and those who have to manage and maintain it. Whether we are dealing with a rural water supply system managed mainly by the user community [60], or a more technically sophisticated urban supply system (see Text S3), this fit is essential. Modern technologies are only manageable if the right skills, resources, and incentives exist, and if appropriate support structures are provided.

### Finance

The level of water sector financing in low-income countries is widely criticised as being inadequate, but at the same time water supply budgets are often underutilised or ineffectively used. Delays in the release of central government funds to local authorities combine with inadequate allocations for operational expenses to render local governments ineffective in disbursing the funds that do reach them. Importantly, though, the additional US$11.3 billion that is needed annually to meet the water and sanitation MDG targets—a relatively small investment (a few dollars per capita per year) that is “highly feasible and within the reach of most nations”—would yield an estimated seven-fold return [39].

Improved water supplies (in JMP terminology) usually attract a tariff or water charge. In low-income countries it is common for such tariffs to be set at levels that are below the real running costs. In such cases a vicious circle often becomes established, in which below-cost tariffs lead to inadequate investment in maintenance, which results in deteriorating service and further unwillingness to pay even low tariffs.

Water consumers without an improved water supply do not pay a financial tariff for water. Even though they may pay heavily in terms of health, time, and energy, it often proves extremely difficult to change the mindset of consumers who are used to water being “free.” Even small water charges are not welcomed by consumers, and revenue collections that start as regular monthly charges often deteriorate to ad hoc collections or disappear altogether. Financial irregularities also often militate against continued payment of charges.

### Strategies to Achieve an Improved Water Supply

Access to a safe and continuous supply of water for drinking, cooking, and personal hygiene is an essential prerequisite for health. An inadequate water supply—whether as a result of poor access or quality, low reliability, high cost, or difficulty of management—is associated with significant health risks. These health risks are experienced most strongly by the poorest nations, and the poorest households within nations. A good water supply is necessary for good sanitation and hygiene, and to underpin livelihoods, nutrition, and economic growth.

The global MDG target on water supply is likely to be met [6] but will leave many hundreds of millions of people without an adequate water supply. Furthermore, the targets are highly unlikely to be met in sub-Saharan Africa. Failure to extend water supply services at an adequate pace is largely a consequence of high population growth rates in the low-income countries, insufficient investment (although the sums needed are not large), and poor governance. Failure of existing water supplies is often due to weak financial and management arrangements for operation and maintenance, and a mismatch between the technology, the water environment, and the capacity of users to maintain systems. The result is poorly performing or broken down urban and rural water supply systems, and continuing poor health.

While the health systems of developing countries are not directly responsible for changing this situation, poor water supplies place large burdens of disease on their populations, and it is those populations and their national health services that pick up the costs of diarrhoea and other diseases. Health professionals should therefore join those from other sectors (infrastructure, education, and economic development) in demanding change.

However, it is clear that many uncertainties remain about how to improve public health through improvements in the water supply. Thus, more and better research is desperately needed, in particular larger and longer double-blinded randomized controlled studies of the health impacts of water supply and quality interventions at the community and household level.

But it is equally clear that action must not wait for the outcomes of such research. We know enough now about the importance of improved water supply, sanitation, and hygiene in relation to health to consider universal access to these services to be an urgent imperative.

## Supporting Information

Text S1 Quantity versus quality and the role of household water treatment.
(0.09 MB PDF)

Text S2 Climate change, drinking water, and health.
(0.07 MB PDF)

Text S3 The growing issue of urban water supply.
(0.07 MB PDF)

## Abbreviations

DALY: disability-adjusted life year
GDP: gross domestic product
JMP: WHO/UNICEF Joint Monitoring Programme for Water-supply and Sanitation
MDG: Millennium Development Goal
NGO: nongovernmental organization
WSP: Water Safety Plan
