# Supplementary material for: Water Supply and Health
Source: PLoS Med. 2010 Nov 9;7(11):e1000361. doi: 10.1371/journal.pmed.1000361 (PMC2976720; doi:10.1371/journal.pmed.1000361)
Supplement: Text S1 — Quantity versus quality and the role of household water treatment. (0.09 MB PDF) [file pmed.1000361.s001.pdf]

Text S1

Quantity versus quality and the role of household water treatment

Arguments over the relative importance of increasing consumption ('quantity') versus improving water safety ('quality') have been taking place since at least the beginning of the first United Nations water decade (the 1980s). For those living distant from a water source, using only around 3-4 litres per person per day, and often suffering from hygiene related disease, the logical priority is to increase quantity consumed, especially for personal and home hygiene. For those at the other end of the spectrum, with water close at hand water quality arguments often carry greater weight.

In the late 1980s Stephen Esrey and colleagues carried out one of the first substantial analyses relevant to this debate [1]. In considering impact on a range of water- and excreta-related diseases, these authors concluded that enhancing access to water (thereby increasing quantity consumed) was more important than improving water quality. More recent systematic reviews came to different conclusions, suggesting that improving drinking water quality at the community and the household level can reduce the incidence of diarrhoeal disease by around 30 to 40% and that this improvement can be seen, even in the absence of other interventions, such as handwashing or sanitation [2,3].

The conclusion of these later reviews has driven many authors and agencies to push for the general uptake of point of use (household) water treatment (HWT), as a major water supply intervention [4]. However, a recent reanalysis of this data has suggested that much of the apparent value of household water treatment may be due to recall bias in unblinded studies and that as yet there is insufficient evidence to justify its widespread promotion [5]. This analysis has led to a strong response from other workers in support of household water treatment [6]. A further systematic review from another independent group cast further doubt on the efficacy of HWT, pointing out problems due to lack of blinding, the lack of an association between diarrhoea and reported compliance, strong evidence of publication bias and potential effects from conflicts of interest [7]. So what is the evidence base for widespread household water treatment and why the confusion?

There are various technologies available for treating water in the home, that include boiling, disinfection or filtration [4]. Disinfection technologies include chlorination with safe storage, commercial, combined coagulant-chlorine systems and SODIS where drinking water is left in clear polyethylene terephthalate (PET or PETE) bottles in the sun to be disinfected by ultraviolet light. The two main types of filter are ceramic (fired clay) pots and the Biosand filter, which is a household version of the slow sand filter.

Evidence in favour of household water treatment:

- There is strong evidence that household water treatment systems are effective at reducing both indicator and pathogen concentrations in drinking water when used properly [8].
- Many randomised trials have suggested a substantial protective effect for acute diarrhoea disease, some of which included objective health outcomes [2,3]. The pooled effect Relative Risk in the most recent analysis (excluding blinded studies) RR=0.51 (95%CI 0.38 – 0.69) [5].

- At least one study has used semi-objective outcome measures (attending medical practitioners) and found a significant effect of HWT [9].
- There is strong evidence for a protective effect against cholera in an outbreak setting [10].
- Many people around the world are apparently using such HWT effectively [6].

Concerns with the evidence base:

- The large majority of these trials have been unblinded using subjective health outcomes and much, but not all of the apparent benefit could be explained by recall bias [5]. In a recent meta analysis of systematic reviews, the authors generated an estimate of the magnitude of due to lack of blinding (ratio of odds ratios 0.75 (0.61 to 0.93)) [11].
- There is strong evidence of publication bias in the published studies of HWT [7,12]
- Most of the reported trials have been of very short duration (<12 months follow-up) and there is evidence that effectiveness declines with time of use [7, 8, 12].
- The evidence that these interventions are used by communities in the long term is not good with continued use being reported as low as 9% for some interventions [4,13].
- Observational studies of diarrhoeal illness rates in communities with high and low water quality have shown only marginal differences in diarrhoeal illness rates [10].

A meta regression analysis of the available data on household water concluded that major part of the heterogeneity in the reported effectiveness of HWT in RCTs is explained by four variables; the technology used, the time between intervention and follow-up, whether or not the study was blinded and whether or not it was conducted in an emergency setting [12]. In particular he concluded that after allowing for the effect of recall bias disinfection technologies such as chlorination or SODIS did not provide any protective effect one year after implementation whereas ceramic filters were associated with a clear reduction in diarrhoeal disease. Given this confusion, how should water supply interventions proceed?

Firstly we would argue that money spent on trials of HWT must be spent on high quality studies, where the evidence can be used with confidence. Short term (<1 year) unblinded studies with subjective outcome measures add little to our understanding of the potential role for household water treatment. Only properly conducted blinded randomised controlled trials with follow-up for two or three years and, where possible, more objective health outcomes will provide the evidence needed.

Secondly there is a need to more adequately consider how HWT be used to best improve public health. Drinking water is one of several transmission pathways for most enteric pathogens and the effectiveness of water quality interventions will depend to large extent on the relative importance of drinking water as a transmission pathway, and also on the extent to which poor hygiene is a consequence of low water availability [66]. In situations where access is adequate, but consumption of faecally-contaminated water is the norm, household water treatment is likely to have significant public health benefits, if ways are found to ensure its sustainability. If households lack reliable

access to adequate water, or personal and home hygiene, food safety and sanitation are poor, the priorities may lie elsewhere.

References

1. Esrey SA, Potash JB, Roberts L, Shiff C (1991) Effects of improved water supply and sanitation on ascaris, diarrhoea dracunculiasis, hookworm infection, schistosomiasis, and trachoma. Bull World Health Organ 69: 609-21.

2. Clasen T, Schmidt WP, Rabie T, Roberts I, Cairncross S (2007) Interventions to improve water quality for preventing diarrhoea: systematic review and meta-analysis. BMJ 334: 782-5.

3. Fewtrell L, Kaufmann RB, Kay D, Enanoria W, Haller L, Colford JM (2005) Water, sanitation, and hygiene interventions to reduce diarrhoea in less developed countries: a systematic review and meta-analysis. Lancet Infect Dis 5: 42-52.

4. Sobsey MD, Stauber CE, Casanova LM, Brown JM, Elliott MA (2008) Point of Use Household Drinking Water Filtration: A Practical, Effective Solution for Providing Sustained Access to Safe Drinking Water in the Developing World. Environ Sci Technol 42: 4261-7.

5. Schmidt W-P, Cairncross S (2009) Household water treatment in poor populations: is there enough evidence for scaling up now? Environ Sci Technol 43: 986-992.

6. Clasen T, Bartram J, Colford J, Luby S, Quick R, Sobsey M (2009) Comment on "Household water treatment in poor populations: is there enough evidence for scaling up now?". Environ Sci Technol 43: 5542-5544..

7. Waddington H, Snilstveit B, White H, Fewtrell L (2009) Water, Sanitation and Hygiene Interventions to Combat Childhood Diarrhoea in Developing Countries. International Initiative for Impact Evaluation (3ie). http://www.3ieimpact.org/admin/pdfs2/17.pdf (accessed July 2010)

8. Arnold BF, Colford Jr JM (2007) Treating water with chlorine at point-of-use to improve water quality and reduce child diarrhea in developing countries: a systematic review and meta-analysis. Am J Trop Med Hyg 76: 354-364.

9. Luby SP, Agboatwalla M, Painter J, Altaf A, Billhimer W, Keswick B, Hoekstra RM (2006) Combining drinking water treatment and hand washing for diarrhoea prevention, a cluster randomised controlled trial. Trop Med Int Hlth 11: 479-489.

10. Gundry S, Wright J, Conroy R (2004) A systematic review of the health outcomes related to household water quality in developing countries. J Water Health 2: 1-13.

11. Wood L, Egger M, Gluud LL, Schulz KF, Jüni P, Altman DG, Gluud C, Martin RM, Wood AJG, Sterne JAC (2008) Empirical evidence of bias in treatment effect estimates in controlled trials with different interventions and outcomes: Meta-epidemiological study. BMJ 336: 601-5.

12. Hunter PR (2009) House-hold water treatment in developing countries comparing different intervention types using meta-regression. Environm Sci Technol 43: 8991-8997.

13. Clasen TF, Brown J, Collin SM (2006) Preventing diarrhoea with household ceramic water filters: Assessment of a pilot project in Bolivia. Int J Env health Res 16: 231-239.

14. Eisenberg J N-S, Scott JC, Porco T (2007) Integrating Disease Control Strategies: Balancing Water Sanitation and Hygiene Interventions to Reduce Diarrheal Disease Burden. Am J Publ Health 97: 846-852.
