# Supplementary material for: Water Supply and Health
Source: PLoS Med. 2010 Nov 9;7(11):e1000361. doi: 10.1371/journal.pmed.1000361 (PMC2976720; doi:10.1371/journal.pmed.1000361)
Supplement: Text S2 — Climate change, drinking water, and health. (0.07 MB PDF) [file pmed.1000361.s002.pdf]

Text S2

Climate change, drinking water and health

It is now widely acknowledged that climate change will have a major impact on the world, and poses one of the major threats to human health and prosperity [1-3]. Increasing levels of greenhouse gases in the atmosphere are predicted to increase global temperature by 2 – 5°C by 2100 [1], although there is growing evidence that both greenhouse gases and temperature are rising faster than these predictions. The consequences of climate change will be much more severe for poorer communities and countries with little ability to adapt. This rise in temperature will affect the water resources and therefore drinking water supplies - but the prediction of impacts is both complex and uncertain. Prediction is uncertain for Africa and Asia, where the lack of data and complicated climate mean that Global Circulation Models cannot accurately replicate past climatic conditions, far less confidently predict future scenarios [1].

Despite the complexity, certain future trends for fresh water resources are predicted with greater confidence: rainfall is likely to be less predictable with increasing frequency of droughts and floods; sea level will continue to rise, leading to salinisation of some coastal aquifers; changes in glaciers and snowmelt in South Asia is likely to lead to long term decreased river flows in sub-catchments where glaciers dominate, although the overall effects on flows in the Indus and Ganges is likely to be more muted [3].So what are the likely impacts on drinking water from these predicted changes to climate?

- There is growing evidence that higher intensity rainfall will lead to greater periodic contamination of water sources, particularly very shallow wells, springs, rivers and ponds [5-7].

- Greater variability in rainfall leads to an increased need for storage of water [8]. This may mean greater investment in dams and piped supplies, or more reliance on the natural storage of groundwater.

- Water supplies for cities and islands reliant on groundwater close to the coast are at increased risk of salinisation, as sea level rises are translated into greater saline ingression into aquifers and river deltas. Salinisation of groundwater due to sea level rise may already be observed in the Ganges-Brahmaputra-Meghna delta [9].

- Extreme weather events such as storms and floods will lead to a greater destruction of water infrastructure, from large city supplies to small community supplies.

- There will be increased competition for water resources, particularly from growing food. Without strong management and protection, the high quality water resources required for drinking may be threatened, particularly for the poorest who have little voice.

- Some technologies will be more resilient to climate change than others [10] Small improved community boreholes equipped with handpumps, that are prevalent over much of Africa are unlikely to fail catastrophically because of climate change [6]. Water sources that are most at risk will be very shallow unimproved sources.

The impact of these changes on human health through effects on drinking water are difficult to predict with certainty, but are likely to impact most heavily on the poorest and those without adequate existing infrastructure [5,11]. Probably the most important impact will be the seasonal loss

of water sources due to aridification, salinisation or failure due to increased demand [11] leading to people turning to even poorer distant water sources. The periodic contamination of existing water sources following heavy rains with pathogens or chemical contaminants could also be a source of disease, as could contamination of surface waters by cyanobacterial blooms [5,12].

References

1. Solomon S , Qin D, Manning M, Chen Z, Marquis M, Averyt KB, Tignor M, Miller HL eds. (2007) Climate Change 2007: The Physical Science Basis. Contribution of Working Group I to the Fourth Assessment Report of the Intergovernmental Panel on Climate Change Cambridge: Cambridge University Press.

2. Bates BC, Kundzewicz ZW, Wu S, Palutikof JP eds. (2008) Climate Change and Water. Technical Paper of the Intergovernmental Panel on Climate Change, Geneva: IPCC Secretariat.

3. Costello A, Abbas M, Allen A et al. (2009) Managing the health effects of climate change. Lancet 373: 1693–1733.

4. Rees GH, Collins DN (2006) Regional differences in response of flow in glacier fed Himalayan rivers to climatic warming. Hydrological Processes 20: 2157-2169

5. Hunter PR (2003) Climate change and waterborne and vector-borne disease. J Appl Microbiol 94: 37S-46S.

6. MacDonald AM, Calow RC, Macdonald DMJ ,Darling WG , Ó Dochartaigh BÉ (2009) What impact will climate change have on rural groundwater supplies in Africa. Hydrolog Sci J 54: 690-703.

7. Taylor, R. G., Miret-Gaspa, M., Tumwine, J., Mileham, L., Flynn, R., Howard, G., Kulabako, R. Increased risk of diarrhoeal diseases from climate change: evidence from urban communities supplied by groundwater in Uganda. In: IASH Publication 334: 15-19.

8. Grey D, Sadoff C (2007) Sink or swim? Watersecurity for growth and development. Water Policy 9: 545-571.

9. Shamsudduha M, Chandler RE, Taylor RG, Ahmed KM (2009) Recent trends in groundwater levels in a highly seasonal hydrological system: the Ganges-Brahmaputra-Meghna Delta. Hydrol Earth Syst Sci 13: 2373-2385.

10. Howard G, Charles K, Pond K, Brookshaw A, Hossain R, Bartram J (2010) Securing 2020 vision for 2030: climate change and ensuring resilience in water and sanitation services. J Water Climate Change 1: 2-16.

11. Calow RC, MacDonald AM, Nicol AL, Robins NS (2010) Ground water security and drought in Africa: linking availability, access and demand. Ground Water 48: 246-256.

12. Hunter PR (1998) Cyanobacterial toxins and human health. J Appl Bacteriol 84 (Suppl): 35S-40S.
