# Supplementary material for: Water Supply and Health
Source: PLoS Med. 2010 Nov 9;7(11):e1000361. doi: 10.1371/journal.pmed.1000361 (PMC2976720; doi:10.1371/journal.pmed.1000361)
Supplement: Text S3 — The growing issue of urban water supply. (0.07 MB PDF) [file pmed.1000361.s003.pdf]

Text S3

The growing issue of urban water supply

Urban water supply provides considerable challenges for public health, particularly in rapidly growing cities. In wealthier countries, the challenges are generally reflected in increased costs for water and tensions between environmental groups, farmers and municipalities. However, for much of the world, increasing urbanisation results in existing municipal supplies becoming overwhelmed. Parts of many cities have no service, and many more have water supplies that are intermittent or otherwise highly unreliable. As a consequence people without reliable service rely on other sources of water at some time or another. Depending on the wealth of the individual this may be bottled water or water from water vendors, surface water or open sewers, shallow wells or deeper boreholes. Furthermore, the drinking water reticulation system may run in close proximity to the sewage and storm water drainage systems. Where sewage collection fails water pipes run through ground contaminated with sewage and grey water. In many poorer urban environments there is an additional problem of illegal access to water in distribution with often unhygienic connections being made further increasing the risk of contamination and risk of disease.

This patchy and unreliable service poses many health challenges. High water pressure within the reticulation network is essential to prevent ingress of pathogens and other contaminants into the supply. Where supplies are intermittent, or where pressure within the pipe falls or supply is interrupted there is a real risk of ingress of pathogens [1]. Indeed, there have been a number of outbreaks in both the developed and developing world that have been attributed to such failures in supply [2,3]. In addition, recent reports have shown that such reduced pressure or supply failure in distribution is associated with increased risk of diarrhoea even in the absence of an identified outbreak [4,5]. Perhaps the starkest indication of the impact on human health of water supply failures comes from the large outbreaks associated with the break-up of the former Soviet Union [6,7], and more recently the outbreak of cholera in Zimbabwe [8].

References

1. LeChevallier MW, Gullick RW, Karim MR, Friedman M, Funk JE (2003) The potential for health risks from intrusion of contaminants into the distribution system from pressure transients. J Water Health 1: 3–14.

2. Hunter, PR (1997). Waterborne disease: epidemiology and ecology. Chichester: Wiley, ISBN 0471-96646-0.

3. Risebro HL, Doria MF, Andersson Y, Medema G, Osborn K, Schlosser O, Hunter PR (2007) Fault tree analysis of the causes of waterborne outbreaks. J Water Health 5 (suppl. 1): 1-18.

4. Hunter PR, Chalmers RM, Hughes S, Syed Q (2005) Self Reported Diarrhea in a Control Group: A Strong Association with Reporting of Low Pressure Events in Tap Water. Clin Infect Dis 40: e32-4.

5. Nygard K, Wahl E, Krogh T, Tveit OA, Bohleng E, Tverdal A, et al. (2007) Breaks and maintenance work in the water distribution systems and gastrointestinal illness: a cohort study. Int J Epidemiol 36: 873–80.

6. Barwick RS, Uzicanin A, Lareau S, Malakmadze N, Imnadze P, Iosava M, Ninashvili N, Wilson M, Hightower AW, Johnston S, Bishop H, Petri WA Jr, Juranek DD (2002). Outbreak of amebiasis in Tbilisi, Republic of Georgia, 1998. Am J Trop Med Hyg 67 :623-31.

7. Mermin JH, Villar R, Carpenter J, Roberts L, Samaridden A, Gasanova L, Lomakina S, Bopp C, Hutwagner L, Mead P, Ross B, Mintz ED (1999) A massive epidemic of multidrug-resistant typhoid fever in Tajikistan associated with consumption of municipal water. J Infect Dis 179: 1416-22.

8. Anonymous (2009) Cholera outbreak, Zimbabwe. Weekly Epidemiol Record 84 (7):50-2.
